# The Treatment of Cholera

**Published:** 1884-11

**Authors:** 


THE TREATMENT OF CHOLERA. By Ch. Gatchell, M. D. Pp. 33; price, 25 cents. Chicago: Gross & Delbridge, 1884.

This little pamphlet tells the story of cholera, its history, its nature its prevention, etc., in a very pleasing style. The book is intended chiefly for the laity; but may be of interest to physicians as well. To the world at large the subject of the *prevention* of epidemics is one of vital interest, and one about which too much cannot be known. Perhaps were our citizens better hygeists they might be more urgent in compelling negligent public servants to do their duty in the matter of city hygiene.

